# Simplified Tools for Measuring Retention in Care in Antiretroviral Treatment Program in Ethiopia: *Cohort* and *Current* Retention in Care

**DOI:** 10.1371/journal.pone.0038555

**Published:** 2012-06-11

**Authors:** Yibeltal Assefa, Alemayehu Worku, Edwin Wouters, Olivier Koole, Damen Haile Mariam, Wim Van Damme

**Affiliations:** 1 Federal HIV/AIDS Prevention and Control Office, Addis Ababa, Ethiopia; 2 Addis Ababa University, School of Public Health, Addis Ababa, Ethiopia; 3 Antwerp University, Department of Social Sciences, Antwerp, Belgium; 4 Institute of Tropical Medicine, Department of Clinical Sciences, Antwerp, Belgium; 5 Institute of Tropical Medicine, Department of Public Health, Antwerp, Belgium; University of California, San Francisco, United States of America

## Abstract

**Introduction:**

Patient retention in care is a critical challenge for antiretroviral treatment programs. This is mainly because retention in care is related to adherence to treatment and patient survival. It is therefore imperative that health facilities and programs measure patient retention in care. However, the currently available tools, such as Kaplan Meier, for measuring retention in care have a lot of practical limitations. The objective of this study was to develop simplified tools for measuring retention in care.

**Methods:**

Retrospective cohort data were collected from patient registers in nine health facilities in Ethiopia. Retention in care was the primary outcome for the study. Tools were developed to measure *“current retention”* in care during a specific period of time for a specific *“ART-age group”* and *“cohort retention”* in care among patients who were followed for the last “Y” number of years on ART. *“Probability of retention”* based on the tool for *“cohort retention”* in care was compared with *“probability of retention”* based on Kaplan Meier.

**Results:**

We found that the new tools enable to measure *“current retention”* and *“cohort retention”* in care. We also found that the tools were easy to use and did not require advanced statistical skills. Both “*current retention”* and *“cohort retention”* are lower among patients in the first two *“ART-age groups”* and *“ART-age cohorts”* than in subsequent *“ART-age groups”* and *“ART-age cohorts”.* The *“probability of retention”* based on the new tools were found to be similar to the *“probability of retention”* based on Kaplan Meier.

**Conclusion:**

The simplified tools for *“current retention”* and *“cohort retention”* will enable practitioners and program managers to measure and monitor rates of retention in care easily and appropriately. We therefore recommend that health facilities and programs start to use these tools in their efforts to improve retention in care and patient outcomes.

## Introduction

Patient retention in care and antiretroviral treatment (ART) has become a critical challenge for HIV care and treatment programs since the last few years [1]–[6]. This is mainly because high levels of retention in care and treatment are related to improved adherence to ART, and as a result, slow progression to AIDS, and increase survival [7]–[9]. It is therefore imperative that health facilities and ART programs measure levels of patient retention in care adequately. Measuring patient retention in care provides practitioners and programme managers with critical information to monitor progress systematically. It can thus help to identify bottlenecks and appropriate interventions related to retention in care; moreover, it facilitates implementation of necessary programmatic changes timely [10].

However, the currently available tools for measuring retention in care, such as Kaplan Meier, have several limitations for adequate program monitoring. The main limitations of these tools are that: (1) the tools need advanced statistical software and analytical skills, which are rarely available at both program and health facility levels, and (2) the tools do not provide current (during a specific period) retention values which are important measures for program monitoring and improvement. Moreover, the rudimentary use of these tools has also other limitations: (3) the tools sum up patients from different years of ART initiation, and (4) the tools merge patients with the same date of enrolment irrespective of the facility where the patients started ART.

**Table 1:** Operational definitions of variables associated with the new tools for measuring retention in care.

Variables	Definition	Numerator	Denominator
**Retention**	All patients who are not registered as deceased or lost to follow-up (LTFU) for any reason	Number of patients alive and on ART	Number of patients alive and on ART plus dead plus LTFU
**Attrition**	This is the opposite of retention, and refers to patients who discontinue care and treatment due to either death or LTFU.	Number of patients who either died or LTFU	Number of patients alive and on ART plus dead plus LTFU
**Loss to follow-up**	Patients who miss scheduled visits to the clinic within three months after the last visit.	Not applicable (NA)	NA
**Transfer out**	It refers to the official transfer of the patient to another clinic	NA	NA
**Transfer in**	It refers to the official transfer of the patient from another clinic	NA	NA
**ART-age**	The number of years that the patient was on ART	NA	NA
**ART-age group**	It refers to the age group that the patient belongs to based on the number of years the patient was taking ART during a specific the patient belongs to based on the calendar	NA	NA
**ART-age cohort**	It refers to the cohort that number of years that the patient was taking ART by the end of a specific calendar	NA	NA
**Current retention**	The retention rate during a specific *“calendar”* among patients who were on ART sometime during the *“calendar”*. The rate can be *“ART-age group”* specific or total.	Number of patients alive and on ART by the end of the calendar	Number of patients alive and on ART by the end of the calendar plus number of patients who died plus LTFU during the calendar
**Cohort retention**	The retention rate by the end of a specific *“calendar”* among a cohort of patients ever started on ART and followed longitudinally overtime. The rate can be *“ART-age cohort”* specific or total. The total *“cohort retention”* is similar to the cumulative retention rate.	Number of patients alive and on ART by the end of the calendar. Note: The numerators for current retention and cohort retention are similar.	Number of patients alive and on ART by the end of the calendar plus number of patients who died plus LTFU ever since patients were started on ART
**Cumulative retention**	The total *“cohort retention”* by the end of the calendar among patients ever started on ART	Number of patients alive and on ART by the end of the calendar	The total number of patients ever started on ART.
**Calendar**	The time during which or by the end of which the retention rate is measured (typically one year, e.g. 2008)	NA	NA
**Probability of retention**	The probability that a patient would be retained after “Y” number of years on ART.	NA	NA

NA: Not applicable.

Many ART programs have therefore initiated a system of “cohort follow-up” which is thought to be usable by practitioners and program managers. This system of “cohort follow-up” defines each group of patients initiated on ART at one site over six months as a separate cohort that will be monitored in parallel with other cohorts. Such system, however, has become very labor intensive after five years of ART delivery in many health facilities and ART programs [11]. Alternatively, some ART programs are monitoring cumulative rates, counting patients “ever initiated on ART” and “currently on ART”, thus yielding retention rate since the start of the program.

All these different tools, including Kaplan Meier, are primarily designed to measure longitudinal retention rates, and do not give information during a specific period (e.g., during the previous six months, 12 months and so on). However, measures for the performance of the health facility or program during a specific period of time are crucial for monitoring and improving retention in care. Hence, in addition to the need for simplified tools that measure longitudinal retention in care, new tools that can provide retention measures during a specified period of time should be developed in order to assess the current performance of a health facility or program. The objective of this study was thus to develop simplified tools, describe these tools and give examples using data from health facilities in Ethiopia.

## Methods

### Ethics Statement

This study was approved by the ethical clearance committee of the Ethiopian Health and Nutrition Research Institute. The data for this study were collected from the routine management information system for patient management and program monitoring. Therefore, patient informed consent was not requested. The Ethics committee was aware of this and approved the secondary use of patient data for this study. We have also got a letter of support from the Federal authorities to collect patient data from the health facilities.

### The antiretroviral treatment program in Ethiopia

A number of initiatives have been undertaken to expand the availability of ART in Ethiopia, including resource mobilization, cost reduction, public-private partnerships, and the public health approach [12], [13]. As a result, ART services have been decentralized and are available in both health centres and hospitals [14]. More than 333,400 and 247,800 patients were “ever started on ART” and “alive and on ART”, respectively, by mid-2011. Retention in care was a challenge for the ART program in the country [4], [6].

### Study design, data collection and analysis

A retrospective cohort study design was conducted in 2009 to determine the outcomes of the ART services in 55 health facilities selected from all regions in the country. Nine health facilities (one tertiary hospital (FH HP), two general hospitals (FS HP and DT HP), two urban health centers (BD HC and GR HC), and four rural health centers (WT HC, BR HC, NM HC and DG HC)), with quite variable rates of retention, were selected among the 55 health facilities for further analysis to identify the reasons for different levels of performance for retention in care.

The data collection for this study was nested within the study, described in the above paragraph, which aimed at identifying the reasons for the different levels of performance. Retention in care was the primary outcome of this study (Table 1). The operational definitions of the different variables used in this study are presented in Table 1. We developed new tools that help to measure retention in care based on the principles used to establish life tables [15], as indicated in Tables 2 and 3. We introduced new concepts or variables such as *“ART-age group”, “ART-age cohort”, “current retention”, “cohort retention”, “calendar”, and “probability of retention*” after ‘y’ number of years on ART (Table 1). We were then able to measure the rates of retention in the nine health facilities using the same principles used for the construction of life tables (Tables 4 and 5) [15]. The *“current”* and *“cohort”* retention rates were then compared across these health facilities. One health facility (FS HP) was selected randomly to check the validity of the new tool (cohort retention) against the retention rate based on Kaplan Meier.

**Table 2:** Calculating *“current retention”* in care stratified by *“ART-age groups.”*

‘ART-Age group’	Death (D)	Loss to follow-up (LTFU)	Number of patients retained by the end of the “calendar” (Ret)	Number of attrition during the “calendar” (Att) = D+LTFU	Proportion of attrition (%Att) = Att/(Ret+Att)	Retention rate (% Ret) = Ret/(Ret+Att)
**<1 year**	D1	LTFU1	Ret1	Att1	%Att1	%Ret1
**1–2 years**	D2	LTFU2	Ret2	Att2	%Att2	%Ret2
**2–3 years**	D3	LTFU3	Ret3	Att3	%Att3	%Ret3
**3–4 years**	D4	LTFU4	Ret4	Att4	%Att4	%Ret4
**4–5 years**	D5	LTFU5	Ret5	Att5	%Att5	%Ret5
**(N–1)-N years**	DN	LTFUN	RetN	AttN	%AttN	%RetN

“Transfer outs” were considered as retained until the date they were transferred out, excluded from the analysis from that date on, and assumed to have similar outcomes as those patients retained by that same date. “Transfer ins” were analyzed with the other patients who were retained by the date of “transfer in”. There were patients who were lost to follow-up (LTFU) for some time, but traced back and had then restarted ART. These patients were considered as “not retained” for the *“calendar”* for which they were LTFU and as “retained” for the *“calendar”* for which they were traced back and had restarted ART.

Table 2 shows how *“current retention”* in care was calculated using data for death (D), loss to follow-up (LTFU) defined as missing scheduled visits to the clinic for more than three months, which occurred during a specific *”calendar”*, and number of patients retained in care (Ret) by the end of the *“calendar”*. D1 represents the number of patients who died during the *“calendar”* among patients in the *“ART-age group”* less than 1 year; similarly D5 represents death among patients in the *“ART-age group”* between 4 and 5 years. %Ret1 is the retention rate during the *“calendar”* among patients in the *“ART-age group”* less than 1 year on ART; %Ret5 is the retention rate among patients between 4 and 5 years on ART. The same logic applies for the other *“ART-age groups”.*

**Table 3:** Calculating *“cohort retention”* in care stratified by *“ART-age cohorts.”*

‘ART-Age cohort’	Death (D)	Loss to follow-up (LTFU)	Number of patients retained by the end of the “calendar” (Ret)	Number of attrition during the “calendar” (Att) = D+LTFU	Proportion of attrition (%Att) = Att/(Ret+Att)	Retention rate (% Ret) = Ret/(Ret+Att)
**<1 year**	D1	LTFU1	Ret1	Att1	%Att1	%Ret1
**<2 years**	D2	LTFU2	Ret2	Att2	%Att2	%Ret2
**<3 years**	D3	LTFU3	Ret3	Att3	%Att3	%Ret3
**<4 years**	D4	LTFU4	Ret4	Att4	%Att4	%Ret4
**<5 years**	D5	LTFU5	Ret5	Att5	%Att5	%Ret5
**<N years**	DN	LTFUN	RetN	AttN	%AttN	%RetN

*Attrition (Att)* was defined as the opposite of retention (Ret). It was calculated as the sum of death (D) and loss to follow-up (LTFU).

Table 3 shows how *“cohort retention”* in care was calculated using data for death (D), loss to follow up (LTFU), which occurred among patients ever started on ART and followed longitudinally ever since they started ART, and the number of patients retained in care by the end of the *“calendar”* (Ret). D1 represents the number of patients who died, from the date of initiation on ART, among patients in the *“ART-age cohort”* less than one year; similarly, D5 represents the number of patients who died, from the date of initiation on ART, among patients in the *“ART-age cohort”* less than five years. %Ret1 is the retention rate by the end of one year on ART; %Ret5 is the retention rate, since initiation on ART, among patients less than five years on ART. The same logic applies for the other *“ART-age cohorts”. “Cohort retention”* is therefore in line with the classic cohort analysis, documenting what has happened to patients, sine the time of initiation on ART, over time.

**Table 4:** *“Current retention”* for a specific *“ART-age group”* during a specific *“calendar”* year ‘Y.’

“ART-Age Group” (A_N_)	Y_1_ (e.g., 2005)	Y_2_ (e.g., 2006)	Y_3_ (e.g., 2007)	Y_4_ (e.g., 2008)	Y_5_ (e.g., 2009)	Y_N_
**A_1 = <1 year_**	CuR_A1_^Y1^	CuR_A1_^Y2^	CuR_A1_^Y3^	CuR_A1_^Y4^	CuR_A1_^Y5^	CuR_A1_^YN^
**A_2 = 1–2 years_**	NA	CuR_A2_^Y2^	CuR_A2_^Y3^	CuR_A2_^Y4^	CuR_A2_^Y5^	CuR_A2_^YN^
**A_3 = 2–3 years_**	NA	NA	CuR_A3_^Y3^	CuR_A3_^Y4^	CuR_A3_^Y5^	CuR_A3_^YN^
**A_4 = 3–4 years_**	NA	NA	NA	CuR_A4_^Y4^	CuR_A4_^Y5^	CuR_A4_^YN^
**A_5 = 4–5 years_**	NA	NA	NA	NA	CuR_A5_^Y5^	CuR_A5_^YN^
**A_N = (N–1)-N_**	NA	NA	NA	NA	NA	CuR_AN_^YN^

NA: Not applicable.

Table 4 shows how *“current retention”* in care was calculated for a specific *“ART-age group”*. *“Current retention”* in care among patients in the *“ART-age group”* ‘A_N_’ during *“calendar”* year ‘Y_N_’, CuR_AN_^YN^, is calculated as the number of patients alive and on ART, Ret, by the end of the *“calendar”* year ‘Y_N_’ divided by the sum of the number of patients alive and on ART by the end of the *“calendar”* year ‘Y_N_’ plus the number of patients who either died (D) or were LTFU during the *“calendar”* year ‘Y_N_’. Hence,

**Table 5:** *“Cohort retention”* for a specific *“ART-age group”* by the end of a specific *“calendar”* year ‘Y.’

‘ART-age cohort’ (A_N_)	Y_1 _(the least recent year)	Y_2_	Y_3_	Y_4_	Y_5_	Y_N_ (the most recent year)
**A_1 = <1 year_**	NA	NA	NA	NA	NA	CoR_A1_^YN^
**A_2 = <2 years_**	NA	NA	NA	NA	CoR_A2_^Y5^	CoR_A2_^YN^
**A_3 = <3 years_**	NA	NA	NA	CoR_A3_^Y4^	CoR_A3_^Y5^	CoR_A3_^YN^
**A_4 = <4 years_**	NA	NA	CoR_A4_^Y3^	CoR_A4_^Y4^	CoR_A4_^Y5^	CoR_A4_^YN^
**A_5 = <5 years_**	NA	CoR_A5_^Y2^	CoR_A5_^Y3^	CoR_A5_^Y4^	CoR_A5_^Y5^	CoR_A5_^YN^
**A_N = <N years_**	CoR_AN_^Y1^	CoR_AN_^Y2^	CoR_AN_^Y3^	CoR_AN_^Y4^	CoR_AN_^Y5^	CoR_AN_^YN^

NA: Not applicable.



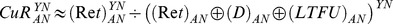



*“Current retention”* thus documents exclusively what happened to patients on ART during a specific *“calendar”* (e.g., 2010). The total *“current retention”* in care during the *“calendar”* year ‘Y_N_’ (CuR_Total_^YN^) was calculated as the total number of patients alive and on ART by the end of year ‘Y_N_’ divided by the total number of patients who died (D) and were LTFU during the *“calendar”* year ‘Y_N_’:
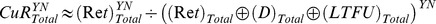



Assuming that the *“current”* retention rates for each *“ART-age group”* A_N_ during *“calendar”* year ‘Y_N_’ in a specific health facility remain the same, the weighted average of one-year retention probabilities (P) of %Ret, weighted by the time since patients started ART, is calculated as:
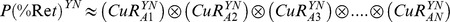



Table 5 shows how *“cohort retention”* in care was calculated. *“Cohort retention”* in care among patients in the *“ART-age cohort”,* ‘A_N_’, by the end of *“calendar”* year ‘Y_N_’, CoR_AN_^YN^, was calculated as the number of patients alive and on ART by the end of the *“calendar”* year ‘Y_N_’ divided by the sum of the number of patients alive and on ART by the end of the *“calendar”* year ‘Y_N_’ plus the number of patients who either died (D) or were LTFU ever since patients started on ART by the end of the *“calendar”* year ‘Y_N_’ among patients in the *“ART-age cohort”* ‘A_N_’. Hence,

**Table 6:** *“Current retention”* in care stratified by *“ART-age group”* in nine health facilities in Ethiopia, in 2009/2010.

Health facility	Retention among different *“ART-age groups”*						Weighted average of one-year retention probabilities
	*“ART-age group”* specific retention					Total	
	0–1	1–2	2–3	3–4	4–5		
**FS HP**	294/313 (94%)	276/298 (93%)	207/208 (99%)	99/99 (100%)	144/144 (100%)	1020/1062 (96%)	86%
**DT HP**	268/279 (96%)	214/248 (86%)	252/273 (92%)	202/211 (96%)	142/148 (96%)	1078/1159 (93%)	70%
**FH HP**	816/920 (89%)	816/886 (92%)	1229/1236 (99%)	1340/1349 (99%)	1238/1238 (100%)	5439/5629 (97%)	81%
**Sub-total for Hospitals**	1378/1512 (92%)	1306/1432 (91%)	1688/1717 (98%)	1641/1659 (99%)	1524/1530 (99.5%)	7537/7850 (96%)	80%
**WT HC**	177/187 (95%)	167/181 (92%)	127/131 (97%)	140/142 (99%)	58/59 (98%)	669/700 (96%)	88%
**BR HC**	190/213 (89%)	159/187 (85%)	143/153 (93%)	138/149 (93%)	61/61 (100%)	691/763 (91%)	66%
**BD HC**	276/287 (96%)	252/270 (93%)	151/157 (96%)	135/138 (98%)	7/7 (100%)	821/859 (96%)	84%
**GR HC**	338/367 (92%)	296/330 (90%)	283/292 (97%)	273/278 (98%)	32/32 (100%)	1222/1299 (94%)	79%
**NM HC**	159/183 (87%)	126/147 (86%)	140/147 (95%)	80/82 (98%)	17/18 (94%)	522/577 (90%)	65%
**DG HC**	169/182 (93%)	146/160 (91%)	102/103 (99%)	118/119 (99%)	57/57 (100%)	592/621 (95%)	83%
**Sub-total for Health Centers**	1309/1419 (92%)	1146/1275 (90%)	946/983 (96%)	884/908 (97%)	232/234 (99%)	4517/4819 (94%)	77%
**Total**	2687/2931 (91.6%)	2452/2707 (90.6%)	2634/2700 (97.6%)	2525/2567 (98.3%)	1755/1764 (99.5%)	12053/12669 (95%)	79.3%

The total *“cohort retention”* in care, also called the *“cumulative retention” in care*, after year ‘Y_N_’ (CoR_Total_^YN^) was calculated as the total number of patients alive and on ART by the end of the *“calendar”* year ‘Y_N_’ (also called currently on ART) divided by the total number of patients who were alive and on ART plus the total number of patients who died (D) and were LTFU from the date of ART initiation until the end of the *“calendar”* year ‘Y_N_’ (also called ever started on ART):

*“Cohort retention”* and *“cumulative retention”* in care are thus indicators for the past and current performance of the health facility. They indicate the performance of the health facility or the program ever since they started ART delivery.

We checked the validity of the tool measuring *“cohort retention”* in care against Kaplan Meier. We used the data from one of the health facilities (FS HP), selected randomly, and compared the estimates, for different years of follow up, based on the new *“cohort retention”* tool with the estimates based on Kaplan-Meier.

Data were collected, between October 2009 and April 2010, from routine patient registries, a hybrid of electronic and paper-based patient management systems, for mortality, LTFU and retention for patients started on ART between September 2005 and August 2010. Data were entered, coded, cleaned and analyzed using SPSS 17 statistical software. Data collection and analysis were conducted by the study staff.

## Results

Table 6 shows that early *“current retention”* in care varies across health facilities, from 87% [81%–91%] in NM HC to 96% [93%–98%] in DT HP and BD HC. It also shows that the difference in *“current retention”* rates narrows as the *“ART-age group”* increases. The weighted average of one-year retention probabilities, based on the current performance of the health facilities, is quite variable, ranging from 65% in NM HC to 88% in WT HC. This variability is mainly due to the difference in early *“current retention”* in care (Table 6); health facilities with better *“current retention”* in care for the first two *“ART-age groups”* are the ones with better weighted average of one-year retention probabilities. The total “current retention” rate is 91.6% and 99.5% in patients with *“ART-age groups”* 0–1and 4–5 years, respectively.

**Table 7:** *“Cohort retention”* in care stratified by *“ART-age cohort”* in nine health facilities in Ethiopia, in September 2010.

Health facility	Retention among different *“ART-age cohorts”*					Total (Cumulative retention rate)
	2009/10 (<1 year)	2008/9 (<2 years)	2007/8 (<3 years)	2006/7 (<4 years)	2005/6 (<5 years)	
**FS HP**	294/313 (94%)	276/323 (85%)	207/295 (70%)	99/221 (45%)	144/243 (59%)	1020/1395, 73%[70–75%]
**DT HP**	268/279 (96%)	214/281 (76%)	252/342 (74%)	202/448 (45%)	142/274 (52%)	1078/1624, 66%[64–69%]
**FH HP**	816/920 (89%)	816/1099 (74%)	1229/1577 (78%)	1340/1873 (72%)	1238/1904 (65%)	5439/7373, 74%[73–75%]
**Sub-total for Hospitals**	1378/1512 (91%)	1306/1703 (78%)	1688/2214 (76%)	1641/2542 (65%)	1524/2421 (63%)	7537/10392, 73%[72–74%]
**WT HC**	177/187 (95%)	167/190 (88%)	127/153 (83%)	140/152 (92%)	58/61 (95%)	669/743, 90%[88–92%]
**BR HC**	190/213 (89%)	159/221 (72%)	143/209 (68%)	138/199 (69%)	61/64 (95%)	691/906, 76%[73–79%]
**BD HC**	276/287 (96%)	252/280 (90%)	151/176 (86%)	135/160 (84%)	7/8 (88%)	821/911, 90%[88–92%]
**GR HC**	338/367 (92%)	296/360 (82%)	283/366 (77%)	274/361 (76%)	32/34 (94%)	1223/1488, 82%[80–84%]
**NM HC**	159/183 (87%)	286/321 (89%)	485/517 (94%)	368/377 (98%)	97/98 (99%)	522/623, 84%[81–86%]
**DG HC**	169/182 (93%)	317/343 (92%)	342/372 (92%)	553/586 (94%)	310/315 (98%)	592/699, 85%[82–87%]
**Sub-total for Health Centers**	1309/1419 (92%)	1477/1715 (86%)	1531/1791 (85%)	1608/1835 (88%)	565/580 (97%)	6490/7392, 88%[87–89%]
**Total**	2687/2931 (92%)	2781/3418 (81%)	3219/4007 (80%)	3249/4377 (74%)	2089/3001 (70%)	14027/17734, 79%[78–80%]

The total *“cohort retention”* in care varied across health facilities, ranging from 66% [64%–69%] to 90% [88%–92%]. Health centers, in general, had higher total *“cohort retention”* in care than hospitals (Table 7). The table also shows that health centers were able to retain a higher proportion of their patients than hospitals for patients who started treatment during 2005/6–2008/9. Health centers also had higher long term retention rates than hospitals; however, there was no difference in early retention in care between health centers and hospitals (Table 7).

**Table 8:** *“Cohort retention”* in care for different *“ART-age cohorts”* in FS HP, Ethiopia, 2005/6–2009/10.

“ART-age Cohort”	2005/6			2006/7			2007/8			2008/9			2009/10			Total		
	Ret	Ret + Att	Ret (%)	Ret	Ret + Att	Ret (%)	Ret	Ret + Att	Ret (%)	Ret	Ret + Att	Ret (%)	Ret	Ret + Att	Ret (%)	Ret	Ret + Att	Ret (%)
**<1 year**	286	326	88	244	297	82	297	348	85	332	357	93	294	313	94	1453	1641	89
**<2 years**	189	227	83	164	213	77	218	254	86	276	298	93	NA	NA	NA	847	992	85
**<3 years**	164	180	91	113	133	85	207	207	100	NA	NA	NA	NA	NA	NA	484	521	93
**<4 years**	149	154	97	99	99	100	NA	NA	NA	NA	NA	NA	NA	NA	NA	248	253	98
**<5 years**	144	144	100	NA	NA	NA	NA	NA	NA	NA	NA	NA	NA	NA	NA	144	144	100

NA: Not applicable.

Table 8 presents the *“cohort retention”* rates for different *“ART-age cohorts”* among patients who started ART in different *“calendars”*, in one of the hospitals, FS HP. We found that the first two *“ART-age cohorts”* had less *“cohort retention”* than the other *“ART-age cohorts”*.

**Figure 1:**
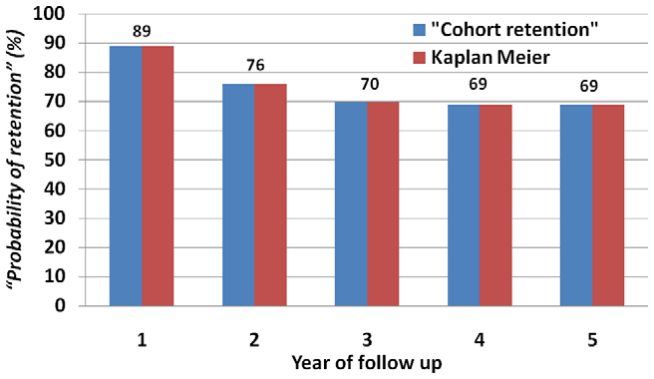
*“Probability of retention”* in care based on *“cohort retention”* and Kaplan Meier in FS HP in Ethiopia, 2010.

**Figure 2:**
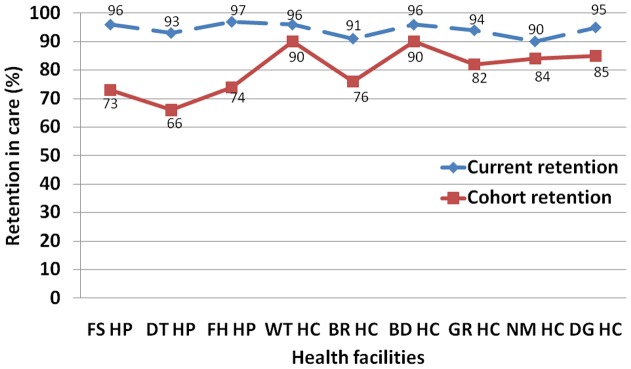
*“Current retention”* and “*cohort retention”* in care in nine health facilities in Ethiopia, mid-2010.

The *“probability of retention”* was estimated, based on the new tool for *“cohort retention”*, for similar *“ART-age cohorts”* from different *“calendars”* (Figure 1). Kaplan Meier was also used to estimate the *“probability of retention”* for different cohorts (Figure 1). The estimates based on Kaplan Meier and *“cohort retention”* were found to be similar (Figure 1). Figure 1 further indicates that *“cohort retention”* in care plummets until two to three years ever since patients started ART, and then stabilizes afterwards.

Figure 2 compares *“current retention” with “cohort retention”*. It shows that *“current retention”* in care is higher than *“cohort retention”* in care across all the nine health facilities. It also shows that the difference between *“current retention”* and *“cohort retention”* in care is wider in hospitals than health centers.

## Discussion

We developed new tools for measuring retention in care in an ART program, based on the principles used for the construction of life tables, which enabled us to measure and compare *“current”* and *“cohort”* retention in care at different times and across nine health facilities in Ethiopia. We ascertained that the tools do not need advanced statistical software and analytical skills, unlike existing tools such as Kaplan Meier. We also found that the estimates based on these new tools were similar to the estimates based on Kaplan Meier (Tables 6 and 7, and Figure 1).

We compared the level of retention of health facilities, and found that *“current”* and *“cohort”* retention rates are variable across health facilities. Health centers, in general, have better *“cohort retention”* rates than hospitals (Tables 6 and 7). The total *“current retention”* rate is lower for *“ART-age groups”* 0–2 years than for *“ART-age groups”* 2–5 years (Table 6). Similarly, we found that the first two years on ART were the most important period for retention in care (Table 7 and Figure 1). This is an indication that the difference in retention rates among health facilities is mainly due to the difference that occurred during the first two years on ART. Hence, health facilities which had better retention in care during the first two years of ART would have better total retention in care (Tables 6 and 7).

We also found that *“current retention”* in care is higher than the cumulative *“cohort retention”* in care; and, the difference between the two is bigger in hospitals than health centers (Figure 2). This might be an indication that hospitals had higher attrition rates in the previous years than health centers. It is also, possibly, because a lot of relatively stable patients were transferred out to health centers from hospitals in the earlier phase of ART delivery [13]. This highlights the different benefits of the indicators *“current retention”* in care and *“cohort retention”* in care. *“Current retention”* in care is an indicator for the current performance of the health facility or the program while *“cohort retention”* in care indicates cumulative performance which combines both the current and previous performances of the health facility or program. Hence, it is important that we define our objectives clearly in order to benefit appropriately from these tools.

Most of the reports and papers published on retention in care are based on either cohort or cumulative estimates. These estimates use tools such as Kaplan Meier and related tools. However, Kaplan Meier and related tools have several inherent limitations. The main limitations of the tools are that (1) the tools need advanced statistical soft ware and analytical skills, which are rarely available at both program and health facility levels, and (2) the tools do not provide current (during a specific period) retention values which are the most important measures for program monitoring and improvement. In addition to these limitations of the tools, the rudimentary use of the tools has other limitations: (3) the tools sum up patients initiated on ART in different years; this is indeed not appropriate in a context where the response is so dynamic and the baseline characteristics of patients are also changing from time to time; moreover, (4) the tools merge patients with the same date of enrolment irrespective of the facility where the patients started ART. It is thus possible that health facilities have high retention rates because they are receiving stable patients from other health facilities. It is also possible that health facilities have low retention rates because they are receiving complex cases from other health facilities. This is indeed a very important limitation of cohort analysis.

The new tools, which were developed to estimate *“cohort retention”* and *“current retention”* in care, address the gaps and avoid the pitfalls associated with the traditional tools and their use. These new tools stratify patients based on their *“ART-age cohort”* and *“ART-age group”*, and estimate retention rates during a specific period of time for each *“ART-age cohort”* or *“ART-age group”*. Contrary to Kaplan Meier and similar tools, one does not need high-level and advanced statistical skills: basic knowledge of excel is sufficient to use the tools for *“current retention” and “cohort retention”. “Current retention”* focuses on the current performance of the health facility or program during a specific period. In general, the added value of these new tools is that the tool for *“cohort retention”* in care simplifies the measurement of retention in care while the tool for *“current retention”* in care provides new and additional information on the health facility's and program’s current performance. This helps health facilities and programs to monitor their performance level and improve patient care and program management.

Our study has both strengths and weaknesses. One of the strengths of the study is that it developed a simplified tool that will be easily usable by program managers and practitioners. The tools can be utilized to monitor the performance of one facility over time, compare one facility with another facility, and learn best practices from those with better retention in care. The second strength of the study is that it developed a tool to measure the current performance of the health facility or the program. It can thus be considered as the first tool measuring *“current retention”*. The third strength of the study is that it described the tools using real data from nine health facilities. The fourth strength of the study is that it estimated retention rates for specific *“ART-age groups”*. This helped us to identify the specific *“ART-age group”* which indeed is crucial for retention in care. Finally, the study checked for the validity of the estimates for the *“probability of retention”* based on *“cohort retention”* in care with the estimates for the *“probability of retention”* based on Kaplan Meier.

The first limitation of the study is that it excluded patients who were “transferred out” and assumed that patients who were “transferred out” had similar outcomes as those who were not. We did not have data to support this assumption in our context. The second limitation of the study was that we did not conduct an explanatory study to find out how health facilities were able to achieve better levels of retention compared to others. However, we have already started other research to identify the outcomes of patients “transferred” out to other health facilities, and to explore the different initiatives implemented by health facilities with better retention in care.

This study has both theoretical and practical relevance for measuring, understanding and improving retention in care. Its theoretical relevance is that the study will indeed improve the existing tools and add knowledge on the measurement of retention in care. Its practical relevance is that it simplifies the measurement of retention in care, and can be applied at both *macro level* (program and policy level) and *meso level* (health facility level) to estimate retention in care and take appropriate action to improve it.

### Conclusion

We developed tools based on demographic principles for the construction of life tables, and described the tools using data from nine health facilities in Ethiopia. We were able to measure and compare *“cohort retention”* and *“current retention”* rates for specific *“ART-age cohorts”* and *“ART-age groups”*, respectively. *“Cohort retention”* rates measure the cumulative performance of a health facility or program while the *“current retention”* rates measure current performance of a health facility or program. These measurements enable practitioners and program managers to monitor their performance, compare one facility with another facility, and learn from relatively better performers to improve patient and program outcomes. Therefore, we recommend that health facilities and programs start to use these tools and explore their practical benefits. We also recommend that a follow up study is conducted to: test the feasibility and acceptability of the new tools to practitioners and program managers, explain how health facilities are achieving high rates of retention in care, and identify the outcomes of patients who were transferred out.

## References

[1] Rosen S, Fox MP, Gill CJ (2007). Patient Retention in Antiretroviral Therapy Programs in Sub-Saharan Africa: A Systematic Review. Plos Med 4(10): e298.. doi:10.1371/journal.pmed.0040298.

[2] Pox MP, Rosen S (2009). Patient retention in antiretroviral therapy programs up to three years on treatment in sub-Saharan Africa: Systematic review.. Tropical Medicine and International Health, 2010.

[3] Tassie JM, Baijal P, Vitoria MA, Alisalad A, Crowley SP (2010). Trends in retention on antiretroviral therapy in National programmes in low-and middle-income countries.. J Acq Immun Def Synd.

[4] Assefa Y, Van Damme W, Haile Mariam D, Kloos H (2010). Toward Universal Access to HIV Counseling and Testing and Antiretroviral Treatment in Ethiopia: Looking Beyond HIV Testing and ART Initiation.. AIDS Patient Care and STDs; 24(8): DOI:10.1089/apc.2009.0286.

[5] Assefa Y, Van Damme W, Hermann K (2010). Human resource aspects of antiretroviral treatment delivery models: current practices and recommendations.. Current Opinion in HIV and AIDS; 5: 78–82; doi:10.1097/COH.0b013e328333b87a.

[6] Assefa Y, Jerene D, Luelseged S, Ooms G, Van Damme W (2009). Rapid Scale-up of Antiretroviral Treatment in Ethiopia: Successes and System-wide Effects. PLoS Med 6(4): e1000056.. doi:10.1371/journal.pmed.1000056.

[7] Hogg RS, Heath KV, Yip B, Craib KJ, O'Shaughnessy MV (1998). Improved survival among HIV-infected individuals following initiation of antiretroviral therapy.. JAMA.

[8] Schilder AJ, Kennedy C, Goldstone IL, Ogden RD, Hogg RS (2001). “Being dealt with as a whole person.” Care seeking and adherence. The benefits of culturally competent care.. Soc Sci Med.

[9] Berg MB, Safren SA, Mimiaga MJ, Grasso C, Boswell S (2005). Nonadherence to medical appointments is associated with increased plasma HIV RNA and decreased CD4 cell counts in a community-based HIV primary care clinic.. AIDS Care.

[10] (2011). WHO, UNAIDS, UNICEF. Towards Universal Access: Scaling Up Priority HIV/AIDS Interventions in the health sector. Progress Report 2010.. Accessed 17 Sept.

[11] (2011). WHO. Country experiences in implementing patient monitoring systems for HIV care and antiretroviral therapy in Ethiopia, Guyana and India: an overview of best practices and lessons learned.. Accessed 17 Sept.

[12] (2011). Ministry of Health of Ethiopia. The single point HIV prevalence estimate.. Accessed 17 Sept.

[13] Assefa Y, Kloos H (2008). The public health approach to ART service scale-up in Ethiopia: the first two years of free ART, 2005–2007.. Ethiop Med J.

[14] (2011). World Health Organization. Treating 3 million by 2005: making it happen: the WHO strategy.. Accessed 17 Sept.

[15] Hsiej JJ (1999). A General Theory of Life Table Construction and a Precise Abridged Life Table Method. Biom.. J.

